# The influence of immune challenges on the mean and variance in reproductive investment: a meta-analysis of the terminal investment hypothesis

**DOI:** 10.1186/s12915-023-01603-4

**Published:** 2023-05-12

**Authors:** Yong Zhi Foo, Malgorzata Lagisz, Rose E. O’Dea, Shinichi Nakagawa

**Affiliations:** Evolution & Ecology Research Centre, School of Biological and Environmental Sciences, University of New South Wales, Sydney, 2052 NSW Australia

**Keywords:** Life-history theory, Phenotypic plasticity, Fecundity compensation, Reproduction, Trade-offs

## Abstract

Finding the optimal balance between survival and reproduction is a central puzzle in life-history theory. The terminal investment hypothesis predicts that when individuals encounter a survival threat that compromises future reproductive potential, they will increase immediate reproductive investment to maximise fitness. Despite decades of research on the terminal investment hypothesis, findings remain mixed. We examined the terminal investment hypothesis with a meta-analysis of studies that measured reproductive investment of multicellular iteroparous animals after a non-lethal immune challenge. We had two main aims. The first was to investigate whether individuals, on average, increase reproductive investment in response to an immune threat, as predicted by the terminal investment hypothesis. We also examined whether such responses vary adaptively on factors associated with the amount of reproductive opportunities left (residual reproductive value) in the individuals, as predicted by the terminal investment hypothesis. The second was to provide a quantitative test of a novel prediction based on the dynamic threshold model: that an immune threat increases between-individual variance in reproductive investment. Our results provided some support for our hypotheses. Older individuals, who are expected to have lower residual reproductive values, showed stronger mean terminal investment response than younger individuals. In terms of variance, individuals showed a divergence in responses, leading to an increase in variance. This increase in variance was especially amplified in longer-living species, which was consistent with our prediction that individuals in longer-living species should respond with greater individual variation due to increased phenotypic plasticity. We find little statistical evidence of publication bias. Together, our results highlight the need for a more nuanced view on the terminal investment hypothesis and a greater focus on the factors that drive individual responses.

**Supplementary Information:**

The online version contains supplementary material available at 10.1186/s12915-023-01603-4.

Given a limited lifespan, individuals face trade-offs between investing in current reproduction vs future reproduction [1–9]. To maximise fitness, individuals are expected to plastically adjust their relative investments in traits that support survival for future reproductive opportunities versus reproducing immediately in response to external and internal environmental cues [10]. An important environmental context that could influence reproductive investment is a survival threat. Two predictions can be made in this situation. On the one hand, individuals might invest in survival (e.g. by redirecting energetic resources to immune function traits [5] at the expense of current reproduction (i.e. reproductive restraint). Doing so could protect future reproductive opportunities (i.e. residual reproductive value) [11]. On the other hand, individuals might adopt the opposite strategy of investing in current reproduction, to make the most of their limited remaining lifespan [11, 12]. This latter prediction has attracted strong theoretical interest, with examples tracing back as early as Fisher’s work from 1930 [13], and has been termed the “terminal investment hypothesis” [14] and “fecundity compensation” [15] (we will use the former term hereafter).

Increasing or decreasing reproduction in response to a survival threat was formerly regarded as discrete strategies (in survival or reproduction). However, an implicit assumption of the terminal investment hypothesis is that the reproductive investment decision exists on a continuum. Whether an individual decreases or increases reproductive investment depends on how much the threat compromises residual reproductive value [13, 16, 17]. This assumption was elaborated recently by the dynamic terminal investment threshold model [17]. When the threat is mild and opportunities for future reproduction are expected (i.e. high residual reproductive value), individuals should prioritize survival by decreasing current reproductive investments. As the threat escalates, residual reproductive value diminishes, and at some point (the terminal investment threshold) it becomes beneficial to switch strategies and maximise current reproductive investment rather than protect survival.

Under the dynamic threshold model, factors that affect an individual’s existing residual reproductive value or their susceptibility to a survival threat should also affect the terminal investment threshold [17]. For example, older individuals have lower residual reproductive value compared to younger individuals [13]. Therefore, older individuals might have a lower threshold at which they terminally invest, compared to younger individuals. Similarly, the perceived magnitude of the threat will depend on individual condition: lower-quality individuals might have a lower terminal investment threshold [17].

Predictions regarding variations in reproductive investment responses have thus far been focused only on changes at the group mean level (Fig. 1). Here, we make the novel prediction that a survival threat might also increase variance within group because the terminal investment threshold is expected to vary across individuals [10, 18]. Provided that the threat is not so severe as to eliminate reproduction or so mild as to elicit no response (i.e. no ceiling or floor effects), we expect varying responses as some individuals decrease (e.g. high quality individuals) while others increase (e.g. poor quality individuals) reproductive investments. This variation in responses should increase population-level variance. This principle of increased phenotypic variance applies to any environmental manipulation that is expected to elicit a phenotypic response and has been empirically demonstrated for responses to limited diet variation (thus limiting chances for individual optimized diet) and high temperature stress [19, 20].

**Fig. 1:**
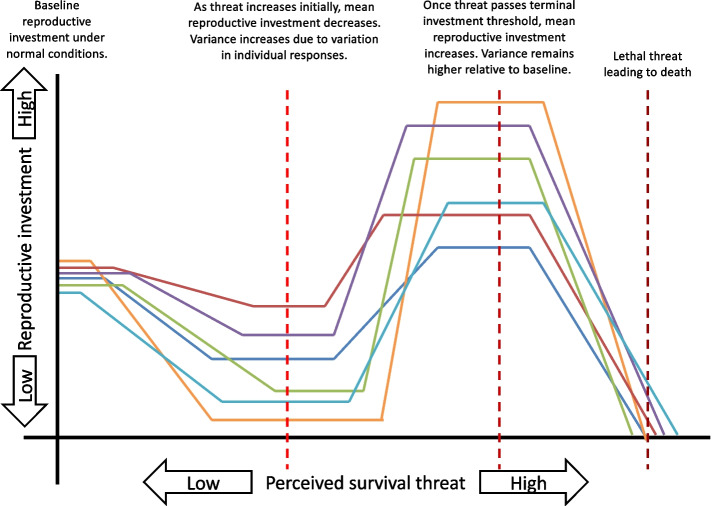
Conceptual model depicting individual terminal investment response varying according to the level of perceived survival threat, leading to potential changes in mean and variance. Each thin continuous line represents a single individual’s reproductive investment as survival threat changes. Vertical dashed lines represent levels of threat where different effects on trait mean or variance can be observed

Despite its prominence, the terminal investment hypothesis has been met with substantial challenges both theoretically and empirically. Theoretically, a recent model posits that the fitness benefits of terminally investing might be more limited than originally thought if lifespan is constrained by somatic damage, rather than time per se [21]. The mathematical model suggests that when we consider the adverse effect of reproduction itself on somatic damages and lifespan, most individuals facing a survival threat would gain greater reproductive benefits by decreasing rather than increasing reproductive investment [21]. Empirically, the hypothesis has attracted scrutiny due to mixed findings. Experimental studies have used a range of manipulations to test the causal effect of a survival threat on reproductive investments, including immune challenges, predator exposure, alarm pheromones, food availability, and somatic damages [17]. While some studies have found support for the hypothesis, others have found null or even opposite results. Together, alternative theoretical models and mixed empirical results call into question the presence or generality of a terminal investment response [17].

Despite the mixed findings, a recent qualitative review by Duffield and colleagues [17] concluded that there was substantial evidence for the terminal investment hypothesis. While noting substantial variation in effects, both across studies and within studies that have measured multiple reproductive traits, Duffield et al. [17] reported that majority of studies found statistically significant evidence (i.e. vote-counting; [22]) for the terminal investment hypothesis.

The review by Duffield et al. [17] also revealed that the most commonly used and well-established experimental paradigm for eliciting a survival threat is to use a non-lethal immune challenge, comprising up to 90% of the experimental studies reviewed. The studies were done on a wide range of taxa, including birds, insects, mammals, reptiles, and amphibians, and measured reproductive traits ranging from mating/courtship behaviours, sexually selected signals/weapons, fertility-related measures, to parental care provisioning. Therefore, studies using this paradigm provide a rich literature with which to evaluate the terminal investment hypothesis.

This paradigm also offers a robust way of testing the terminal investment hypothesis with the use of non-live immune challenges (e.g. by using a non-live substrate, such as dead pathogens, or non-pathogenic particles, like nylon). If the studies used live pathogens (and parasites), the effect of the threat might be confounded by the negative impact of the pathogen on health. If sick individuals have a lower reproductive output than healthy individuals, it does not necessarily mean they have strategically decreased reproductive effort; they might have increased effort within their limited capacity while trying to recover from the ill effects of the infection. Non-live immune challenges avoid this conundrum because they do not actually compromise survival. Using non-live substrates also avoids confounds due to live pathogens manipulating the hosts. For example, a landmark paper using this approach, by Bonneaud and colleagues [16], induced an immune response in female breeding house sparrows using a vaccine against the Nobi-Vac Paramyxo virus. Vaccinated females were more likely to lay replacement clutches and produce heavier and larger nestlings, providing support for the terminal investment hypothesis.

Here, we aim to build on the qualitative review by Duffield et al. [17] with a quantitative one. We perform a systematic review and meta-analysis to examine the predictions that a non-lethal immune challenge increases mean reproductive investments in iteroparous animals (Fig. 2). To better understand why inconsistent effects are found in the literature [17], we then test for moderators of the overall mean effect. We also examine the hitherto-untested hypothesis that an immune challenge would increase variability in reproductive investments, as derived from the dynamic threshold model, using recent developments in meta-analytic methods for analysing group differences in variance [23]. Our list of potential moderators and predictions is provided in Table 1 for the mean and variance effects, respectively. As this is a registered report, the inclusion of a particular moderator in our analysis is subject to whether there are sufficient sample sizes in our final dataset; see “Methods” section for details.

**Fig. 2:**
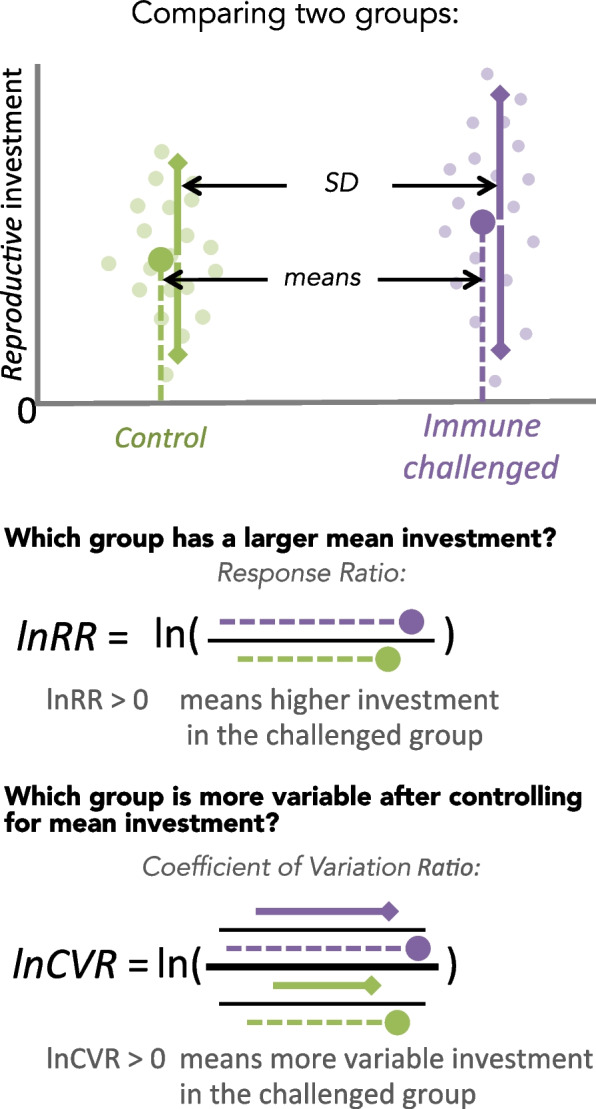
Our main predictions represented as individual study result (a dot plot) with the expected differences in mean and variance between the control and treatment (immune challenged) group. Details on the calculations and interpretations for our mean and variance effect sizes are presented below the dot plot

**Table 1:** Predictions for potential moderators for mean and variance terminal investment effect (note that the inclusion of a particular moderator in our analysis is subject to whether there are sufficient sample sizes in our final dataset; see “Methods” section for details)

Moderator	Predicted mean effect	Predicted variance effect	Rationale for predictions
Average lifespan of species	Shorter-living species > longer-living species	Longer-living species > shorter-living species	**Mean prediction:** Longer-living species are likely to have greater future reproductive prospects compared to shorter-living species. Therefore, they might exhibit a lower terminal investment effect **Variance prediction:** Longer-living species have greater phenotypic plasticity in general [1]. Therefore, we expect the variance effect to be greater in longer-living species
Age class	Older > Younger	Mixed age > Young or old	**Mean prediction:** Compared to younger individuals of the same species, a given survival threat is more likely to tip older individuals past their terminal investment threshold and cause them to increase reproductive investments [13]. There is some evidence supporting this prediction (e.g. [24]) **Variance prediction:** We expect mixed age samples to contain individuals in different life-history stages. Therefore, such samples will show a greater variance in reproductive investment responses compared to more homogenous samples. We do not have any specific predictions for young versus old individuals because there are many potential situations that can influence differences in variance between the two groups. Young categories may include short- and long-lived individuals, whereas old classes only long-lived ones. In such cases, the variance will be greater in the young individuals. Alternatively, young individuals may all lean towards investing less in current reproduction in favour of future reproductive prospects. Here, we might see a smaller variance in younger individuals
Whether focal sex provides extended parental care	No extended parental care > Extended parental care	No specific predictions	**Mean prediction:** Individuals with extended parental care might be less inclined to invest in reproduction during a simulated infection because of the possibility of transferring potentially harmful infections to the offspring [17]
Control procedures used	Control procedures used that do not invoke an immune response > control procedures used that might invoke an immune response (e.g. via wound healing through sham surgical implants or injections)	No specific predictions	**Mean prediction:** Some control procedures may inadvertently invoke an immune response via wound healing [16] and consequently cause the control individuals to upregulate their reproductive investments. One example is sham implants, where the control group is subject to all the surgical procedures of an implant except that the implant does not contain the active test ingredient. The lacerations sustained during the sham procedure may invoke an immune response via wound healing. We expect the terminal investment effect size to be smaller in such studies
Experimental setting (Laboratory versus wild experiments)	No specific prediction	No specific predictions	**Mean prediction:** We might expect the terminal investment effect to be stronger in laboratory studies, where confounding variables can be better controlled. Alternatively, wild individuals might be more likely to show a terminal investment response due to their higher mortality rates (i.e. lower terminal investment threshold) [25] **Variance prediction:** Populations brought into the laboratory are likely to face more homogenous environmental conditions during the experiment. Depending on whether the laboratory environment was suited to the needs of all individuals in the sample, we may observe reduced or increased variation in the response to the immune challenge
Source of the animals (wild species, wild-caught and bred in the lab for several generations, and cultured commercial species)	No specific predictions	No specific directional predictions	**Variance prediction:** Individuals from the wild might be more varied due to environmental differences. Therefore, they might show more variance in their responses to the immune challenge. Alternatively, wild populations might already have hit a ceiling in their phenotypic variability. In this case, we might expect the more homogenous populations (e.g. those that have been bred/cultured for several generations) to respond with increased variance
Reproductive investment categories (pre-mating sexually selected physical traits, mating/courtship behaviours and effort, parental care provisioning, reproductive output, later-life offspring traits, and post-copulatory traits)	No specific predictions	No specific predictions	
Type of immune challenge (non-pathogenic foreign bodies versus substrates of pathogenic origins)	No specific predictions	No specific predictions	
Taxonomic group	No specific predictions	No specific predictions	

## Methods

We prepared our protocol in accordance with the Preferred Reporting Items for Systematic Review and Meta-Analysis Protocols (PRISMA-P) statement [26], where applicable (some items were not applicable because PRISMA-P is customized for medical systematic reviews).

### Eligibility criteria

The overall description of our study based on the PICO framework [27] is as follows:

- Population = non-human animal species without any history of artificial selection, including mutants
- Intervention = non-lethal immune challenge via experimental treatment
- Comparison/Control group = unchallenged group of animals, otherwise in the same state
- Outcome = reproduction-related traits

To elaborate, we included experimental studies that report the effect of a non-lethal immune challenge on reproduction-related traits in non-human, non-laboratory, and non-domesticated adult metazoan animals. Human populations were excluded due to potential confounds of modern medicine on reproduction. Populations that are known to have undergone artificial selection, such as laboratory model strains (e.g. laboratory mice and rats) and domesticated species (e.g. dogs and cats), were excluded, so that the conclusions generalized to natural conditions. Plant populations were excluded because their immune systems and life-history strategies can be very different from animals. The experiments must contain at least two conditions: (1) immune-challenge treatment with non-live agents or substances that are expected to trigger an immune response without being lethal (e.g. dead pathogens or their parts, exogenous proteins or other substances) and (2) normal/untreated or control treatment (e.g. placebos that trigger, at most, a weak immune response, such as a saline injection). For the immune-challenge treatments, we excluded active infections of any kind due to the possibility of host manipulation by the live parasites/pathogens. The experimental group must not be subject to additional manipulations (e.g. special diet, stress) that could affect immune responses or reproduction.

We included eligible studies from all years available from our information sources (detailed below). Languages that our research lab members could read, and were therefore retained, included English, Mandarin, Japanese, Slavic languages, German, and Indonesian. Both published and unpublished (i.e. grey) literature (e.g. unpublished manuscripts and academic theses) were included.

### Information sources

First, we conducted the literature search on two published literature databases, Web of Science and Scopus. Second, we located relevant papers from the reference lists of major qualitative reviews and from papers that cited these reviews. Third, we searched for grey literature using the databases, ProQuest EBSCO, OpenGrey, and the search engine Google Scholar (Google Scholar indexes both published and unpublished work. We used it to identify unpublished work only). We included eligible studies from all years, subject to the coverage limits of each of the sources and language restrictions outlined above.

### Search strategy

On two occasions, 23/01/2020 and 02/07/2022, we searched titles, abstracts, and keywords in Web of Science, Scopus, ProQuest, and EBSCO using the following Boolean search strings.

#### Web of Science

TS = ( ( "terminal investment" OR "reproductive effort" OR "fecundity compensation" OR "reproductive compensation" OR "reproductive fitness" OR "reproductive investment" OR "reproductive success" OR "Life History Trade-Off*" OR "Phenotypic* Plastic*" OR "pre-copulatory NEAR/5 trait*" OR "sexual NEAR/5 weapon*" OR "sexual NEAR/5 ornament*" OR "post-copulatory NEAR/5 trait*" OR "ejaculate quality" OR "sperm quality" OR "mating effort" OR "parental care") AND ( "immune challeng*" OR "immunochalleng*" OR "infect*" OR lipopolysaccharide OR lps OR phytohemagglutinin OR pha OR "sheep red blood cells" OR srbc OR implant* OR vaccin* OR nylon OR sephadex)) NOT TS = ( load OR human OR people OR men OR women OR infant* OR rat OR rats OR mouse OR mice OR pig* OR pork OR beef OR cattle OR sheep OR lamb* OR chicken* OR calf* OR horse* OR infective).

#### Scopus

TITLE-ABS-KEY ( ( "terminal investment" OR "reproductive effort" OR "fecundity compensation" OR "reproductive compensation" OR "reproductive fitness" OR "reproductive investment" OR "reproductive success" OR "Life History Trade-Off*" OR "Phenotypic* Plastic*" OR "pre-copulatory W/5 trait*" OR "sexual W/5 weapon*" OR "sexual W/5 ornament*" OR "post-copulatory W/5 trait*" OR "ejaculate quality" OR "sperm quality" OR "mating effort" OR "parental care") AND ( "immune challeng*" OR "immunochalleng*" OR "infect*" OR lipopolysaccharide OR lps OR phytohemagglutinin OR pha OR "sheep red blood cells" OR srbc OR implant* OR vaccin* OR nylon OR sephadex)) AND NOT (load OR human OR people OR men OR women OR infant* OR rat OR rats OR mouse OR mice OR pig* OR pork OR beef OR cattle OR sheep OR lamb* OR chicken* OR calf* OR horse* OR infective).

#### ProQuest

( ( "terminal investment" OR "reproductive effort" OR "fecundity compensation" OR "reproductive compensation" OR "reproductive fitness" OR "reproductive investment" OR "reproductive success" OR "Life History Trade-Off*" OR "Phenotypic* Plastic*" OR "pre-copulatory NEAR/5 trait*" OR "sexual NEAR/5 weapon*" OR "sexual NEAR/5 ornament*" OR "post-copulatory NEAR/5 trait*" OR "ejaculate quality" OR "sperm quality" OR "mating effort" OR "parental care") AND ( "immune challeng*" OR "immunochalleng*" OR "infect*" OR lipopolysaccharide OR lps OR phytohemagglutinin OR pha OR "sheep red blood cells" OR srbc OR implant* OR vaccin* OR nylon OR sephadex)) NOT ( load OR human OR people OR men OR women OR infant* OR rat OR rats OR mouse OR mice OR pig* OR pork OR beef OR cattle OR sheep OR lamb* OR chicken* OR calf* OR horse* OR infective).

#### EBSCO

( ( "terminal investment" OR "reproductive effort" OR "fecundity compensation" OR "reproductive compensation" OR "reproductive fitness" OR "reproductive investment" OR "reproductive success" OR "Life History Trade-Off*" OR "Phenotypic* Plastic*" OR "pre-copulatory N5 trait*" OR "sexual N5 weapon*" OR "sexual N5 ornament*" OR "post-copulatory N5 trait*" OR "ejaculate quality" OR "sperm quality" OR "mating effort" OR "parental care") AND ( "immune challeng*" OR "immunochalleng*" OR "infect*" OR lipopolysaccharide OR lps OR phytohemagglutinin OR pha OR "sheep red blood cells" OR srbc OR implant* OR vaccin* OR nylon OR sephadex)) NOT ( load OR human OR people OR men OR women OR infant* OR rat OR rats OR mouse OR mice OR pig* OR pork OR beef OR cattle OR sheep OR lamb* OR chicken* OR calf* OR horse* OR infective).

For Google Scholar, due to the limited Boolean search functionality, we used the search terms “terminal investment” AND “reproduction” AND “immune challenged” | “immune challenge””. Google Scholar orders results based on relevance. For efficiency, we screened the top 50 results from each year.

For OpenGrey, due to the limited number of publications in this database, we used the search term “immune challenge”, which allowed us to screen through any publications relevant to immune challenges.

### Study records and selection process

The screening and selection process was done in two stages. First, we conducted an initial screening of the titles and abstracts of the retrieved bibliometric records to exclude papers that clearly did not meet our inclusion criteria. The bibliometric records were uploaded to the online literature screening application Rayyan [28]. After removing duplicates, two authors, YZF and ML, independently screen all titles and abstracts. A decision tree was created and piloted prior to screening. Screening decision conflicts were resolved via a discussion until agreement between the two reviewers. Second, we conducted full-text screening to identify papers meeting all our inclusion criteria. Two reviewers, YZF and ML, did the full-text screening independently using a pre-determined and piloted standardized form derived from our inclusion/exclusion criteria (see “Eligibility criteria” section). Conflicts were resolved via a discussion and agreement between the two reviewers.

Following the identification of the eligible full-texts, data extraction was split among the four authors and done using a pre-determined and piloted standardized form. The resultant dataset was then checked by two of the authors, SN and ML. Inconsistencies were resolved via discussion among the authorship team. Some publications contained duplicated or partially duplicated datasets (e.g. thesis data and published version of the same dataset). In such cases, we retained the version that had a greater sample size and/or reported more information for us to derive our meta-analytic statistics or determine our moderator variables. In cases of that contained multiple control and/or treatment groups in the same study, we extracted the data from all of them for moderator analyses. We attempted to contact authors of papers published within the last 5 years to request for missing/necessary data.

### Data items

#### Effect size calculations

We analysed the effects of treatment on mean and variance separately. For the mean effect size, we used the natural logarithm of the response ratio (*lnRR*) between the means of the control and the immune challenge groups [23, 29] (Fig. 2). We used *lnRR* for our main analysis rather than the standardized mean difference (Hedges’ *d*), because it represents the differences in means independent of a linear difference in variance between groups. However, as part of sensitivity analyses, we also analysed the data in the supplementary material using Hedges’ *d* [30], which increased our sample size as it could be calculated using non ratio-scale data and could be calculated from inferential statistics when descriptive statistics were missing (e.g. *t*, *F*, and *p*-values with sample size or degrees of freedom).

For the variance effect size, we used the natural logarithm of the ratio between the coefficients of variation (*lnCVR*) [23] of the control versus the immune challenge groups (Fig. 2). We required the means, sample sizes, and dispersions to calculate *lnCVR*. Accurate estimation of *lnCVR* required a larger sample size than *lnRR*. Therefore, we also analysed the overall effect for variance using a second method that afforded more statistical power in the supplementary material [23]. We used the natural logarithm of the standard deviation (*lnSD*) with treatment versus control groups and the natural logarithm of mean reproductive investment (*lnMean*) as the predictor variables (see details below).

#### Moderators

Potential moderators to the mean and/or variance effects and how they were coded are listed below. We have three categories of moderators: study-related, species-related, and publication-related moderators. We prioritized obtaining the relevant information from the publication itself. If the information was not reported, we obtained it from other published sources or databases such as AnAge [31] or Animal Diversity Web (https://animaldiversity.org/), where applicable (e.g. for species-related or publication-related moderators).

*Study-related moderators*

**Residual reproductive value.** We approximated residual reproductive value by classifying age as a categorical variable (e.g. young, middle-aged, old, and mixed age categories) based either on the authors’ classifications or by comparing the age information provided in the paper with the reproductive lifespan of the species. Given the age distribution of the samples in our dataset was mostly clustered around the first half of the respective species’ lifespans and then across the third and fourth quarters, samples that are less than half the reproductive age were classified as young, more than half to three quarter as middle-aged, and more than three quarter as old.

**Reproductive investment categories.** Reproductive investment can be assessed using a wide range of measures. Our initial list included seven categories: (1) Reproductive output; (2) Offspring traits; (3) Parental care provisioning; (4) Mating/courtship behaviour and effort; (5) Pre-mating physical trait; (6) Post-copulatory trait; (7) Others. Following a discussion among the authors, to ensure sufficient effect sizes in each categories, we re-coded our list into five broader categories:

1. Reproductive output: these are typically related to female fecundity (e.g. the number of eggs and offspring produced, the total mass of eggs, egg size, the success rate of reproduction, hatching success of eggs)
2. Offspring traits and success: these are characteristics of offspring (e.g. the size of offspring or the total mass of offspring, or their fledging success, or the success rate to independence)
3. Behavioural traits: these include parental care provisioning behaviours (e.g. feeding, incubating, and related behaviours) as well as pre-mating or during-mating behaviours that increase the chance of successful mating and fertilization (such as mate guarding)
4. Physiological/Physical traits: these include morphological traits that are associated with pre-copulatory sexual selection (e.g. male-male competition or female choice) and primary sexual characteristics (formerly known as “Post-copulatory trait”) such as reproducing organs (testes and ovaries) and related traits (e.g. sperm number and seminal vesicle weight)
5. Others: these are traits that are not immediately obvious to classify to any of the levels above, for example, latencies to breeding or mating, or egg protein level or yolk volume in relation to egg size

**Type of infection.** Typical immune challenges employed in the evolutionary literature include (1) foreign benign implants such as nylon; (2) sheep red blood cells (SRBC); (3) phytohemagglutinin (PHA) challenge; (4) lipopolysaccharide (LPS; outer membrane of bacteria) challenge; (5) vaccination; (6) innoculated parasitic challenges (non-live or extracts); (7) others [32]. We categorized the challenges into either non-pathogenic foreign bodies versus substrates of pathogenic origins.

**Laboratory versus wild experimental testing conditions.** Studies were categorized into those that were conducted in a laboratory setting versus those conducted in the wild, as reported in the original studies.

**Source of the species.** In order to ensure that our results generalize to natural conditions, we chose to exclude any populations that have been artificially selected (see details above). However, some of the included populations may experience genetic changes inadvertently without deliberate artificial selection, such as wild-caught animals that were maintained in a facility for several generations. Therefore, we coded our samples into wild species, wild-caught, and bred in the lab for several generations and cultured commercial species.

**Type of control group used.** The control group procedures were classified into those that might invoke an immune response (e.g. sham implants or placebo injections) versus those that would not (e.g. no additional procedures).

**Blinding.** To test for potential bias due to experimenters being privy to treatment conditions assigned to the test individuals, we coded studies based on whether experimenters were blinded (yes, no, uncertain/not reported).

**Selective reporting.** We tested for potential bias due to selective reporting of results based on whether the papers presented all results in an extractable format (e.g. all relevant descriptive and inferential statistics) or not (e.g. omitted due to non-significant results or inadequate details reported, such as *p* < 0.05) [27, 33]. There are other types of selective reporting, such as omitting dependent variables that were initially included in registered protocols [27]. We chose to focus on inadequate reporting of results because, in our experience, it is the most common type of selective reporting in this field.

*Species-related moderators*

**Taxon.** Species were categorized into their broad taxonomic groups, such as mammals, birds, fishes, reptiles/amphibians, and insects.

**Lifespan of the species.** We coded the lifespan of the species based on the recorded average lifespan (in days), which were gathered from a range of sources, including databases (AnAge, Animal Diversity Web), published peer-review journal articles, government, and other websites.

**Whether the focal sex provides extended parental care.** We classified the species into those in which the focal sex provides parental care versus those in which the focal sex does not provide parental care.

*Publication-related moderators*

**Year of publication.** Year of publication was recorded, to examine potential time lag bias (i.e. tendency for studies with large effects to be published earlier [34]).

**Journal impact factor.** Journal impact factor (Journal Citation Reports, 2017) of published papers at the year of publication was coded for analysing potential bias in journals, particularly those with high-impact factors, to publish findings with stronger effects [35].

### Outcomes and prioritization

As defined in our PICO statement above, our outcome measurements were reproduction-related traits (pre-mating sexually selected physical traits, mating/courtship behaviours and effort, parental care provisioning, reproductive output, later-life offspring traits, and post-copulatory traits), which were measured in response to the experimental immune challenge. Thus, we included all potential reproductive investment traits for which our main effect sizes, namely *lnRR* and *lnCVR*, could be calculated.

### Risk of bias in individual studies

We assessed risk of bias in individual studies by coding the information on blinding and selective reporting, as described above.

### Data synthesis

All data were quantitatively summarized using multilevel meta-analytic models [36] (see Additional file 1 for the dataset and Additional file 2 for the R analysis code). We ran our meta-analyses using the *rma.mv* function in the R package *metafor* [37]. We controlled for potential non-independence in the dataset using four potential random effects: study ID, paper ID, species ID, and phylogeny (using the phylogenetic correlations matrix); we also used an observation-level (effect size level) random effect. Phylogeny was created using the *R* package *rotl* [38], based on data from the Open Tree of Life database [39]. We decided on the final random effect structure upon considering the model fit (*I*^2^), the biological relevance of particular combinations of random effects, and the number of levels in each random effect. When multiple variables are measured on the same group of animals, not only are the measurements non-independent (i.e. phenotypically correlated), but their sampling errors are also non-independent. To address this issue and obtain the correct variance estimates, we used robust variance estimation with study ID as the clustering level.

We tested for overall mean and variance effects using intercept-only meta-analytic models. We then computed *I*^2^ to test whether there was substantial heterogeneity in the effect sizes. Following Senior et al. [40], we presented total *I*^2^, *I*^2^ due to each random effect, and the estimated variance components (i.e. *τ*^*2*^). If the total *I*^*2*^ was medium or above (i.e. 50% or above) [41], we proceeded to conduct moderator (meta-regression) analyses. The moderator analysis results may be unreliable if particular levels of a moderator were over-represented in our dataset. Therefore, prior to our analysis, we examined the sample sizes within each moderator to ensure that that the ratio of the smallest number of cases to the largest was no less than 1:10. In cases that exceeded the 1:10 ratio, we first tried to balance the ratio by combining some of the levels. When that was impossible, we discarded that particular moderator. If there was any potential multicollinearity between moderators, we either combined the moderators into a single one or drop some of the moderators from our analyses. For missing data (effect size and/or moderators), we chose to conduct the moderator analyses on the complete cases only [42, 43], or drop a given moderator from analyses.

For the moderator analyses (i.e. meta-regression analyses), first, we considered univariate models with each moderator. Second, the full set of study-related, species-related, and publication-related moderators, except journal impact factor (note impact factor will only be available for published articles), were entered into AICc-based model selection and averaging procedure using the R package *MuMin* [44]. A list of models containing all the possible combinations of moderators was generated. The “best” model, defined as the model with the lowest AICc value, was identified together with all models that were within 2 AICc points from the “best” model. We then averaged the coefficients of the models identified to generate the final averaged model results. For all meta-regression analyses, we calculated their marginal *R*^2^ [45].

For the *lnSD* analyses, we examined the overall effect of treatment group (referred to in the formula below as Group) on *lnSD* while controlling for the natural log of the mean reproductive investment. Here is an example of this model using the notation suggested by Wilkinson and Rogers [46]:

$$\mathrm{lnSD }\sim 1 +\mathrm{ Group }+\mathrm{ lnMean }+ (1 +\mathrm{ Group}|\mathrm{ study\ ID}) + (1 |\mathrm{ species\ ID}) \dots$$

Note that the regression coefficient for Group is comparable to the meta-analytic (overall) mean from models with *lnCVR*. We included the same random effects that were modelled for *lnCVR*.

### Meta-bias(es)

Potential bias was assessed based on whether AICc model selection retained the moderators blinding, selective reporting, or year of publication, and whether they significantly moderated the mean and variance effects. Potential bias due to journal impact factor (tendency for higher impact factor journals to publish studies with large effects) was tested separately on the data published in journal articles. Here, we entered journal impact factor as a predictor together with moderators retained by our AICc model selection to test whether impact factor predicts effect size.

We tested for publication bias for the mean effect using a variant of Egger’s regression test [47], which accounts for non-independence among effect sizes [48]. We entered the square root of the inverse of the effective sample size of effect sizes (i.e. the square root of sampling variance) as an additional predictor into the final model from our AICc model selection. If this predictor was significant, we then entered the inverse of the effective sample size in replacement and tested whether adjusted effect size at the inverse of the effective sample size = 0 was significant. We also repeated this Egger’s test using a univariate model with SE/variance as the only predictor of effect size.

### Confidence in cumulative evidence

We assessed confidence in our results by qualitatively interpreting information from (1) meta-analysis and meta-regression (i.e. overall means, their confidence intervals and the degree of heterogeneity and how such heterogeneity was explained by moderators, especially those that indicate risk of bias, e.g. complete reporting) and (2) publication bias and sensitivity analyses (i.e. whether these analyses indicate potential biases).

### Departure from registered protocol

We note the following changes from the registered protocol, all of which arose due to either logistical difficulties, improved statistical software, or changes to recommended methods after the acceptance of our Stage 1 registration. All changes were decided prior to our statistical analyses with the exception of departure numbers 6 and 7, which were performed following reviewer feedback during Stage 2 full manuscript review.

1. The number of papers we collected was more than double what we expected. Therefore, it was too laborious for two reviewers to extract the data independently in duplicate as planned originally. Instead, data extraction was divided between all authors, and underwent a separate round of cross-checking for quality assurance.
2. We had planned originally to run the analysis for *lnSD* using *MCMCglmm* because it enables us to incorporate random slopes into multilevel models. But we were able to do the same using an updated version of *metafor*. Therefore, we ran all analysis using *metafor*.
3. Instead of assuming a 0.5 correlation between sampling errors of repeatedly measured individuals and entering the covariance matrix into our meta-analytic models to control for non-independence in sampling errors, we used robust standard errors with sample ID as the cluster variable. This newer method does not require us to make any assumptions about the correlation between sampling errors but instead derives it directly from the dataset [49, 50].
4. Instead of the standard Egger’s regression test, we tested for publication bias using a recently developed variant of this test that has been shown to be more sensitive at detecting publication bias than the original [48].
5. Upon further discussion, we decided to conduct the publication bias analyses only on the mean and not the variance effect. Traditionally, the field of biology has been primarily concerned about mean changes. Therefore, we do not expect biases towards effects involving changes in variance [51].
6. Lifespan data of species can be confounded by the total research effort spent on a given species [52]. Therefore, to control for this confound, we include total research effort for that species (defined as the number of independent entries per species in the Scopus database) as a covariate whenever lifespan is included in a model (sensu [53]).
7. Following comments from reviewers during Stage 2 full-text submission, we conducted additional sensitivity analyses using a subset of data from the two largest taxonomic groups in our dataset, namely birds and insects.

## Results

### Dataset description

**Fig. 3:**
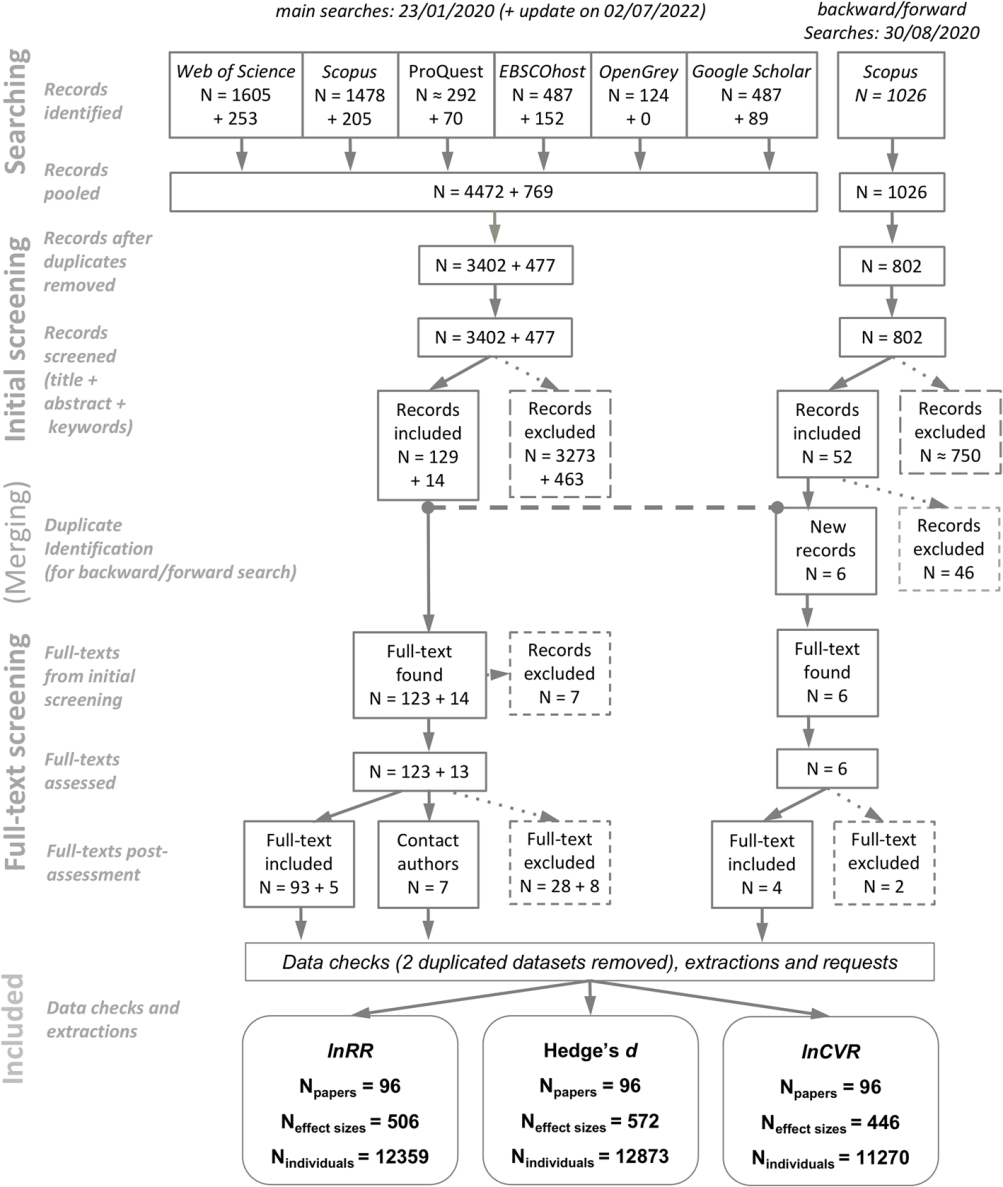
PRISMA diagram of the literature search process and the final number of included papers, effect sizes, and individuals by effect size used

**Fig. 4:**
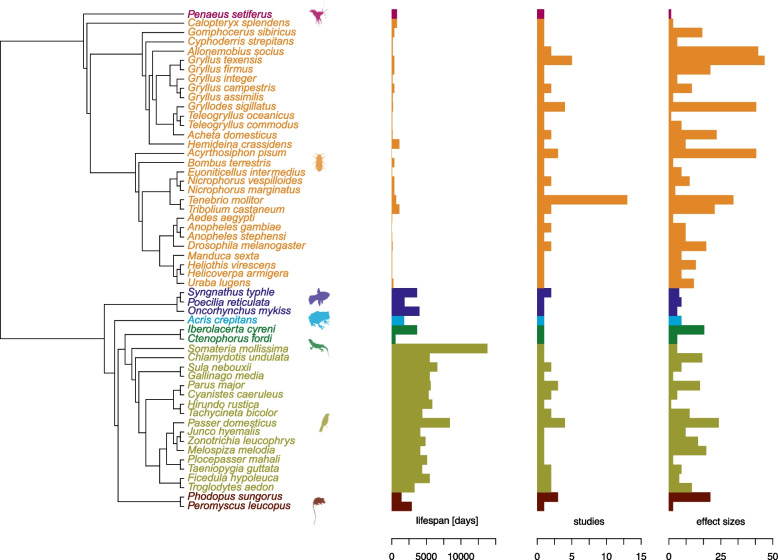
Phylogenetic tree of our overall dataset, including the lifespan, number of studies, and number of effect sizes for each species

We summarized the literature search process using the PRISMA diagram [26] in Fig. 3. We had a total of 96 papers which provided, depending on the type of effect size used, 446 to 572 effects sizes arising from the data of 11,270 to 12,873 individuals (see Fig. 3 for details). The dataset contained 54 species from taxonomic groups including crustaceans, insects, fishes, amphibians, reptiles, birds, and mammals (Fig. 4).

The final list of moderators included age class, control group immune response, population category, reproductive category, treatment type, blinding, incomplete reporting, species lifespan, journal impact factor (for meta-analysis of means only), and year of publication (for meta-analysis of means only). Moderators were included only when (1) There were sufficient cases at each level, such that the ratio of the smallest number of cases to the largest was no less than 1:10; and (2) moderators were not substantially correlated (i.e. multicollinearity). As per our registered protocol, to satisfy the first criteria, age class and reproductive category were re-coded by combining the levels into broader categories. The final levels of each moderator can be found in Tables 3 and 5 below.

There were strong associations among four of the moderators that were decided a priori, namely taxonomic group, parental care, lifespan, and experimental setting, where birds and mammals were more likely to be long living and to provide parental care. Birds were also more likely to be studied in the wild. See Supplementary Figure S1 (Additional file 3) for details of the associations. Therefore, to satisfy the second criterion of avoiding multicollinearity, we retained lifespan, which had the strongest theoretical impetus as a moderator, and dropped the other three moderators.

### Overall effect of immune challenge on mean reproductive investment response

**Fig. 5:**
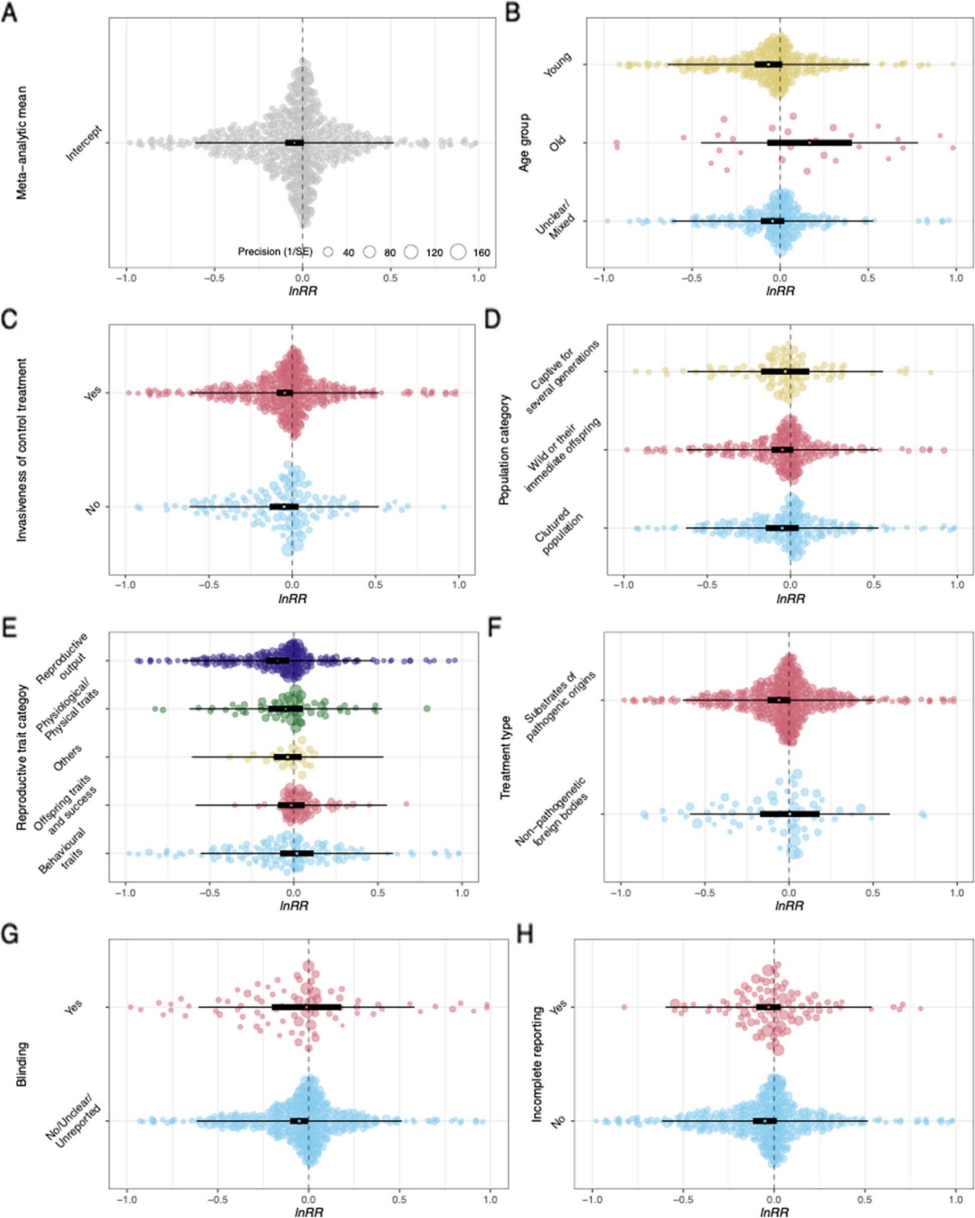
Orchard plots [54] for the **A** overall effect size and **B–H** categorical moderator effects for mean reproductive investment responses to an immune challenge. Positive *lnRR* indicates increased reproductive investment in the treatment group. Each plot includes the mean effect size (open circle), 95% confidence interval (thick error bars around the mean effect size), 95% prediction interval (thin error bars), and the distribution of individual effect sizes (with the size of the points corresponding to their precision). *X* axes were truncated at − 1 and 1 to improve the visibility of the summary plots (i.e. mean point estimates, confidence intervals, and prediction intervals). See supplementary figure S2 (Additional file 3) for untruncated versions of the plots

Among the five potential random effects shown in Table 2, paper ID, study ID, and observation ID accounted for a substantial proportion of the heterogeneity and were therefore retained in all meta-analytic models testing the mean response. Overall, there was no effect of an immune challenge on mean reproductive investment, *lnRR* =  − 0.05, *p* = 0.09, 95% CI (− 0.10, 0.01) (Table 3; Fig. 5).

**Table 2:** Heterogeneity among the mean effect sizes by potential random effects

	*I*^2^
Total	96.75%
Paper ID	53.05%
Study ID	10.33%
Observation ID	33.99%
Species ID	0.17%
Phylogeny	0.00%

**Table 3:** Parameter estimates, *p*-values, and marginal *R*^2^ for the effect of an immune challenge on mean reproductive investment. *M* is the mean *lnRR* effect size (positive value indicates increased reproductive investment for the treatment group), CI.lb and CI.ub are the lower and upper bounds of the 95% confidence interval. ^a^Indicates moderators that were retained in the AICc final averaged model

	*M*	*P*	CI.lb	CI.ub	marginal *R*^2^
**Meta-analytic mean**	− 0.046	0.086	− 0.099	0.007	
**Age class**^**a**^					
Unclear/mixed	− 0.043	0.205	− 0.110	0.024	0.037
Old	0.167	0.171	− 0.073	0.407	
Young	− 0.066	0.097	− 0.144	0.012	
**Control procedure**					
No	− 0.049	0.268	− 0.136	0.038	0.000
Yes	− 0.045	0.074	− 0.094	0.004	
**Source of animals**					
Cultured population	− 0.050	0.319	− 0.148	0.049	0.001
Wild or immediate offspring of wild	− 0.049	0.136	− 0.114	0.016	
Wild-caught but kept in research facilities for generations	− 0.032	0.661	− 0.176	0.112	
**Reproductive investment categories**^**a**^					
Behavioural traits	0.019	0.713	− 0.081	0.118	0.029
Offspring traits and success	− 0.015	0.712	− 0.095	0.065	
Others	− 0.036	0.386	− 0.119	0.046	
Physiological/physical traits	− 0.048	0.358	− 0.151	0.055	
Reproductive output	− 0.097	0.007	− 0.167	− 0.027	
**Immune challenge type**^**a**^					
Non-pathogenic foreign bodies	0.005	0.955	− 0.172	0.182	0.006
Substrates of pathogenic origins	− 0.060	0.086	− 0.127	0.009	
**Blinding**^**a**^					
No/unclear	− 0.052	0.040	− 0.102	− 0.002	0.003
Yes	− 0.012	0.900	− 0.204	0.179	
**Incomplete reporting**^**a**^					
No	− 0.050	0.129	− 0.116	0.015	0.001
Yes	− 0.030	0.367	− 0.096	0.036	
**Log(lifespan of species) (mean centred and controlling for research effort for that species)**	− 0.010	0.756	− 0.068	0.048	0.002
**Journal impact factor (mean centred)**	0.011	0.557	− 0.026	0.048	0.001
**Year of publication (mean centred)**^**a**^	− 0.039	0.136	− 0.090	0.012	0.018

### Effects of moderators on mean responses

**Fig. 6:**
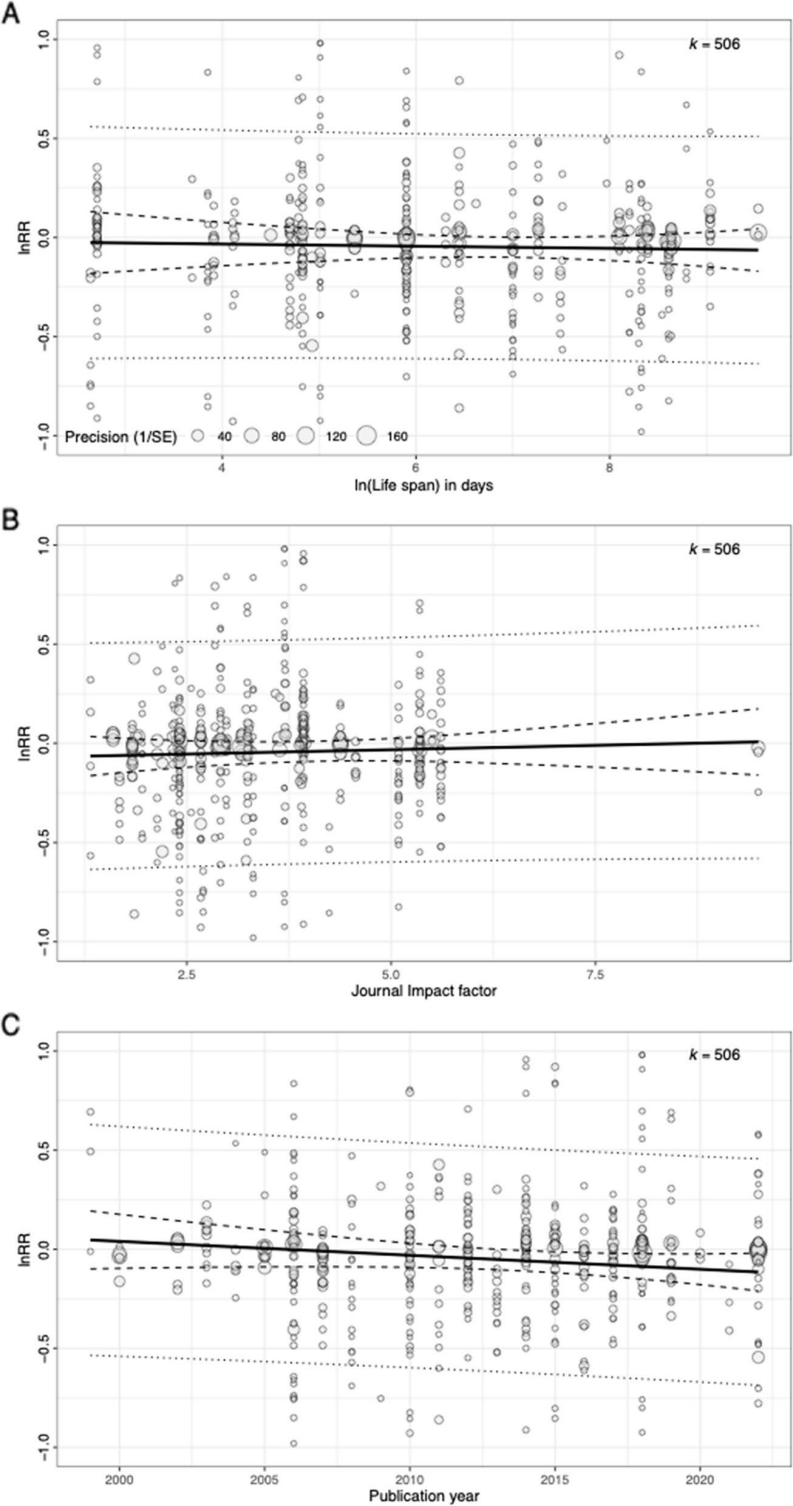
Relationship between effect size and lifespan, publication journal impact factor, and publication year for mean reproductive investment responses to an immune challenge. Size of each point corresponds to the precision (inverse of standard error). *k* refers to the number of effect sizes. *Y* axes were truncated at − 1 and 1 to improve the visibility of the summary plots (i.e. fitted line, confidence intervals, and prediction intervals). See supplementary figure S3 (Additional file 3) for untruncated versions of the plots

Given the large heterogeneity among the effect sizes, *I*^2^ = 96.75%, we conducted moderator analyses. The final averaged model from the AICc model selection included six moderators: age class, reproductive category, treatment type, year of publication, blinding, and incomplete reporting (Table 3, Figs. 5 and 6). Out of these six moderators, age class and reproductive category showed an effect. In terms of age class, old individuals showed a positive effect (i.e. a terminal investment response; Table 3, Fig. 5) that was significantly different from young individuals, estimate = 0.22, *p* = 0.02, 95% CI (0.03, 0.40), or mixed age individuals, estimate = 0.20, *p* = 0.04, 95% CI (0.01, 0.39). For reproductive category, reproductive output had a negative effect (Table 3, Fig. 5) that was significantly different from behavioural traits, estimate = 0.13, *p* = 0.01, 95% CI (0.03, 0.23). The rest of the four moderators did not show any significant effects (*p*-values ranging from 0.17 to 0.70). Overall, the moderator analysis showed that older individuals are more likely to terminally invest than younger individuals while reproductive output is likely to decrease in face of an immune challenge.

### Overall effect of immune challenge on variance in reproductive investment

**Fig. 7:**
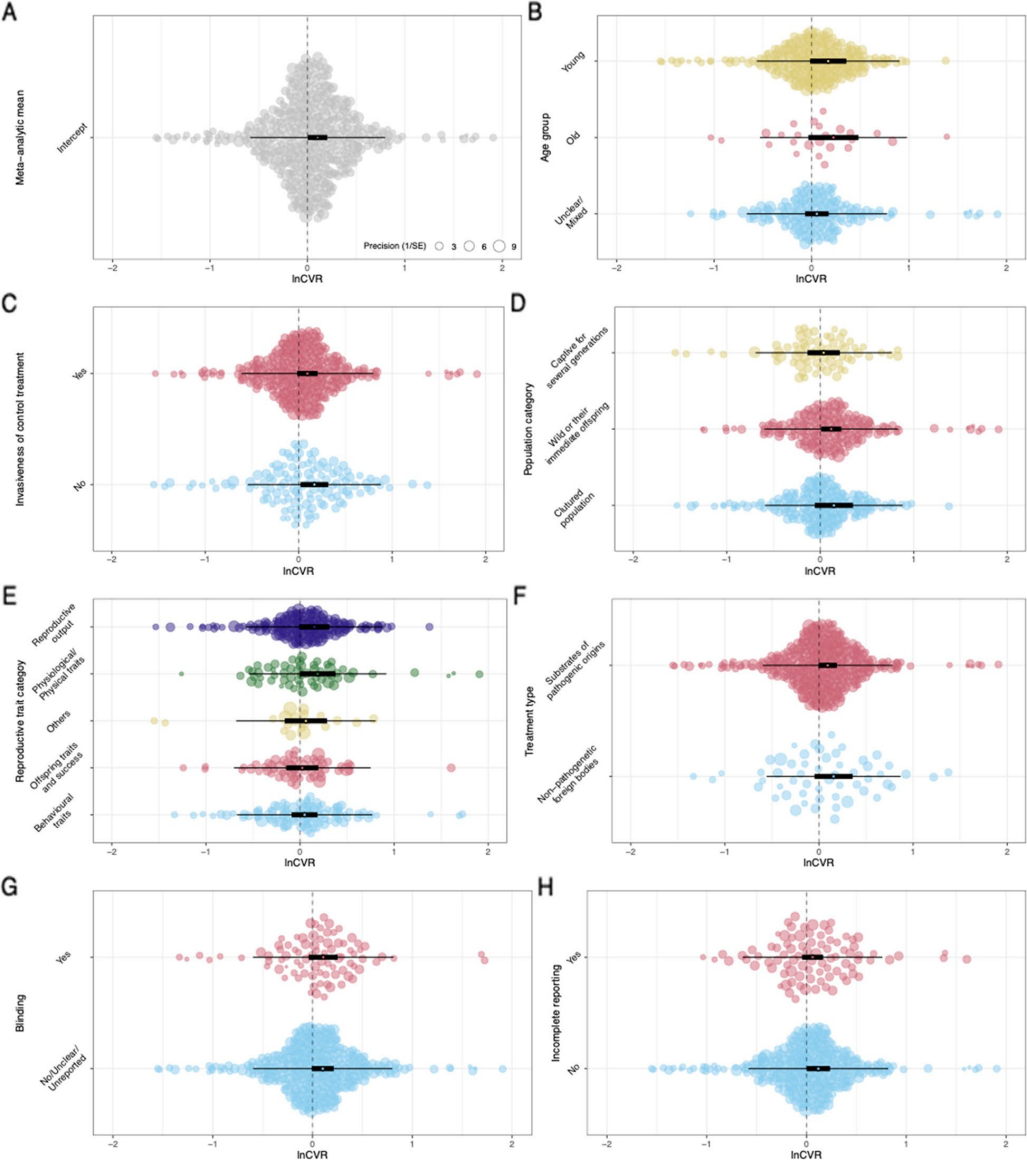
Orchard plots for the **A** overall effect size and **B–H** categorical moderator effects for variance in reproductive investment responses to an immune challenge. Positive *lnCVR* indicates increased variance in the treatment group. Each plot includes the mean effect size (open circle), 95% confidence interval (thick error bars around the mean effect size), 95% prediction interval (thin error bars), and the distribution of individual effect sizes (with the size of the points corresponding their precision). *X* axes were truncated at − 2 and 2 to improve the visibility of the summary plots (i.e. mean point estimates, confidence intervals, and prediction intervals). See supplementary figure S4 (Additional file 3) for untruncated versions of the plots

We included all random effects except species ID in all meta-analytic models testing the variance effect because they each accounted for a substantial proportion of the heterogeneity in effect sizes (Table 4). Overall, individuals showed an increase in variance following an immune challenge relative to the control group, *lnCVR* = 0.10, *p* = 0.04, 95% CI (0.00, 0.20) (Table 5, Fig. 7).

**Table 4:** Heterogeneity among the variance effect sizes by potential random effects

	*I*^2^
Total	65.33%
Paper ID	22.20%
Study ID	7.58%
Observation ID	26.75%
Species ID	0.00%
Phylogeny	8.81%

**Table 5:** Parameter estimates, *p*-values, and marginal *R*^2^ for the effect of immune challenge on variance in reproductive investment. *M* is the mean *lnCVR* effect size (positive value indicates increased variance in the treatment group), CI.lb and CI.ub are the lower and upper bounds of the 95% confidence interval. ^a^Indicates moderators that were retained in the AICc final averaged model

	*M*	*p*	CI.lb	CI.ub	marginal *R*^2^
**Meta-analytic mean**	0.104	0.040	0.005	0.2034	
**Age class**					
Unclear/mixed	0.054	0.381	− 0.067	0.175	0.028
Old	0.225	0.087	− 0.033	0.482	
Young	0.171	0.072	− 0.016	0.357	
**Control procedure**^**a**^					
No	0.167	0.028	0.018	0.316	0.009
Yes	0.093	0.097	− 0.017	0.202	
**Source of animals**					
Cultured population	0.145	0.162	− 0.059	0.350	0.011
Wild or immediate offspring of wild	0.117	0.033	0.009	0.224	
Wild-caught but kept in research facilities for generations	0.036	0.683	− 0.137	0.209	
**Reproductive investment categories**^**a**^					
Behavioural traits	0.049	0.488	− 0.089	0.186	0.028
Offspring traits and success	0.024	0.782	− 0.147	0.195	
Others	0.062	0.585	− 0.162	0.287	
Physiological/Physical traits	0.187	0.054	− 0.003	0.376	
Reproductive output	0.152	0.065	− 0.009	0.313	
**Immune challenge type**^**a**^					
Non-pathogenic foreign bodies	0.155	0.134	− 0.048	0.358	0.004
Substrates of pathogenic origins	0.093	0.063	− 0.005	0.190	
**Blinding**^**a**^					
No/unclear	0.103	0.066	− 0.007	0.212	0.000
Yes	0.108	0.144	− 0.037	0.253	
**Incomplete reporting**^**a**^					
No	0.118	0.048	0.001	0.235	0.004
Yes	0.060	0.261	− 0.046	0.166	
**Log(lifespan of species) (mean centred and controlling for research effort for that species)**^**a**^	0.087	0.023	0.012	0.162	0.064

### Effects of moderators on variance in responses

**Fig. 8:**
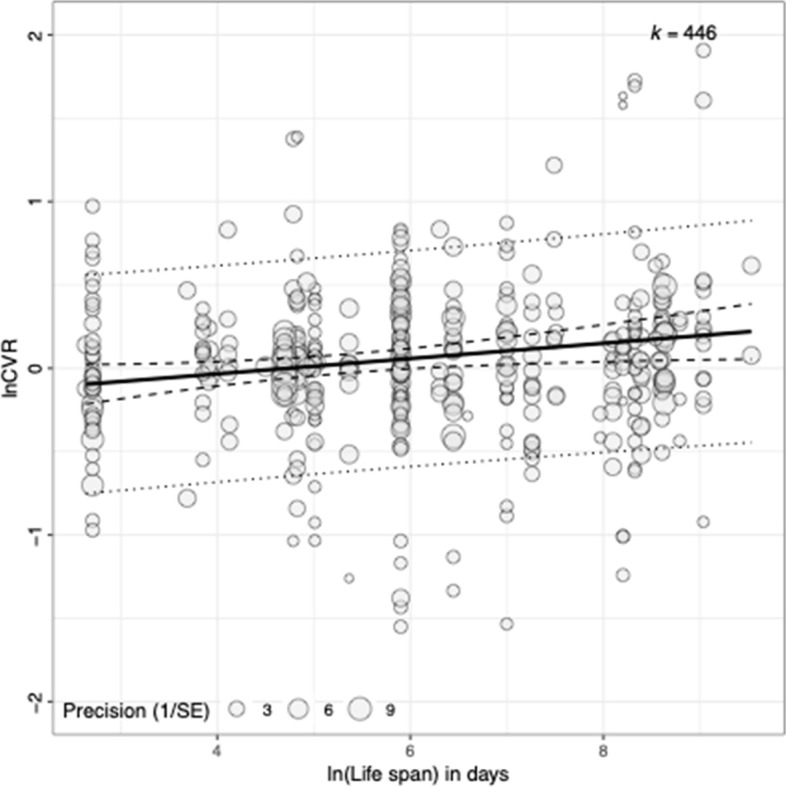
Relationship between effect size and lifespan for variance in reproductive investment responses to an immune challenge. Size of each point corresponds to the precision (inverse of standard error). *k* refers to the number of effect sizes. *Y* axis was truncated at − 2 and 2 to improve the visibility of the summary plot (i.e. fitted line, confidence interval, and prediction interval). See supplementary figure S5 (Additional file 3) for untruncated version of the plot

Given the medium-to-large overall heterogeneity among the effect sizes, *I*^2^ = 65.33%, we conducted moderator analyses. The final averaged model from the AICc model selection included four moderators: species lifespan, control group immune response, incomplete reporting, and treatment type (Table 5, Figs. 7 and 8). Out of these four moderators, there was only a statistically significant effect of lifespan, where longer-living species respond to an immune challenge with a larger increase in variance of reproductive investment relative to shorter-living species, slope = 0.09, *p* = 0.01, 95% CI (0.02, 0.16) (Fig. 8). In other words, longer-living species show a greater variation in response to an immune challenge.

### Publication bias

**Fig. 9:**
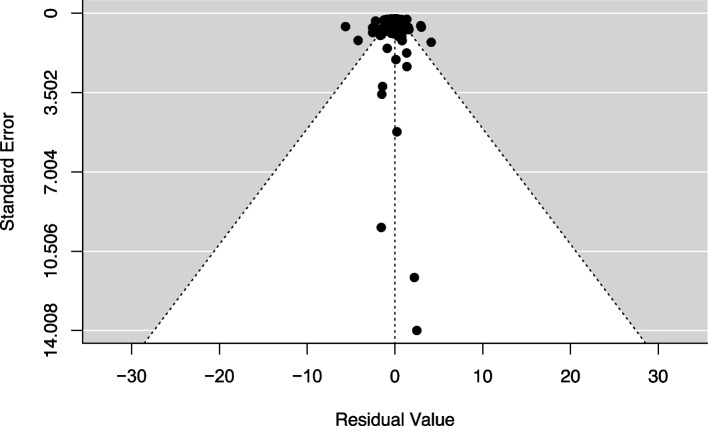
Funnel plot of mean effect residuals after controlling for the moderators that were retained by our AICc model selection, plotted against the standard error. Visual inspection found no asymmetry in the distribution of the residuals, suggesting that there is no evidence that indicate publication bias

We found no evidence of potential publication bias for the mean effect sizes. Visual inspection of the funnel plot did not reveal any potential funnel plot asymmetry (Fig. 9). This lack of asymmetry was corroborated by our variant of Egger’s test, which did not find any significant slope for level of uncertainty, both when entered as a single predictor: slope =  − 0.24, *p* = 0.20, 95% CI (− 0.59, 0.12), and with the other moderators that were retained by the AICc model selection: slope =  − 0.26, *p* = 0.18, 95% CI (− 0.65, 0.12). Similarly, we did not detect any time lag bias, journal impact factor bias, or small study effects. Year of publication was retained in the final averaged model from our AICc model selection, but the slope was not significant: slope =  − 0.04, *p* = 0.17, 95% CI (− 0.09, 0.02). Journal impact factor did not predict effect size: slope = 0.005, *p* = 0.86, 95% CI (− 0.05, 0.06). Altogether, these results suggest that our meta-analytic findings are robust.

### Sensitivity analyses using Hedges’ d and lnSD

Hedges’ *d* and *lnSD* results are presented in the supplementary results section (Additional file 3). To summarize, there were two main differences in these supplementary analyses. Hedges’ *d* analysis did not find a statistically significant difference in the mean response between old and young individuals, even though the direction of the overall effect was the same. *lnSD* analysis failed to find an increase in variance in the treatment group.

### Sensitivity analyses on data from birds and insects

Some of the findings, such as the effect of age on mean changes and the effect of lifespan on variance changes, can be due to differences both within and across taxonomic groups. Given that taxonomic order could not be incorporated into our models with the other moderators due to multicollinearity concerns, we decided to examine the impact of order by conducting additional analyses with a subset of our data from the two orders with the most effect sizes: birds and insects. First, we compared the results between birds and insects to check for differences in responses across order. Then we ran the moderator analyses for each order separately.

#### Mean responses

There was no significant difference in mean changes between birds and insects, estimate = 0.034, *p* = 0.62, 95% CI (− 0.099, 0.17). For the moderator analyses in birds, we could only include reproductive category, immune challenge type, blinding, incomplete reporting, lifespan, and year of publication. Age group, control procedure, and source of animals could not be included because the distribution of effect sizes were too heavily skewed towards young and wild populations that were tested with control procedures that might trigger an immune response. The final averaged model from the AICc model selection included two moderators: blinding and year of publication. There was an effect of blinding, where effects involving experimenters that were blinded to the experimental conditions were significantly smaller than those that were not, estimate =  − 0.17, *p* = 0.001, 95% CI (− 0.27, − 0.07).

We were able to include all moderators for the insect data. The final averaged model from the AICc model selection included four moderators: age class, immune challenge type, blinding and year of publication. There was a significant effect for age class, where old individuals showed a significantly more positive effect than young individuals, estimate = 0.18, *p* = 0.03, 95% CI (0.01, 0.34). Overall, the subset results were in agreement with our findings from the full dataset.

#### Variance responses

There was a marginally significant difference in variance changes between birds and insects, estimate =  − 0.11, *p* = 0.06, 95% CI (− 0.22, 0.01), with birds showing a significant increase in variance following an immune challenge, estimate = 0.12, *p* = 0.02, 95% CI (0.02, 0.21), while insects did not, estimate = 0.01, *p* = 0.75, 95% CI (− 0.04, 0.06). For the moderator analyses in birds, we could only include reproductive category, treatment type, blinding, incomplete reporting, lifespan, and year of publication, similar to the mean analyses above. The final averaged model from the AICc model selection included five moderators: blinding, species lifespan, immune challenge type, incomplete reporting, and year of publication. There was an effect of blinding, where effects involving experimenters that were blinded to the experimental conditions showed a significantly greater change in variance than those that were not, estimate = 0.25, *p* = 0.01, 95% CI (0.06, 0.44). There was also an effect of lifespan, where longer-living species showed a stronger increase in variance than shorter-living species, estimate = 0.14, *p* = 0.0004, 95% CI (0.06, 0.22).

We were able to include all moderators for the insect data. The final averaged model from the AICc model selection included five moderators: control procedure, source of animals, immune challenge type, blinding, and incomplete reporting. There was only a marginally significant effect for control procedure, where effects using controls that potentially triggered an immune response showed a smaller change in variance compared to those using controls that do not, estimate =  − 0.09, *p* = 0.05, 95% CI (− 0.18, 0.0003). Overall, the subset results showed that the effect of lifespan in our overall findings were due to differences both between taxonomic groups and within.

## Discussion

Since first described in the 1930s [13], the terminal investment hypothesis has remained controversial as decades of empirical research continue to produce equivocal findings. To quantitatively summarize these disparate findings, we conducted a meta-analysis of studies testing the experimental effect of a simulated immune challenge on reproductive investment. Our results showed that the way individuals invest in reproduction following an immune challenge is highly nuanced. Not only was there very high heterogeneity in effect sizes across studies, but individuals within study also diverged in their responses, leading to a variance increase in the treatment group. Our results also revealed moderator factors that are responsible for such varied responses. On average, older individuals have a stronger tendency to terminally invest (i.e. increase reproductive investment) compared to younger individuals. In terms of variance, longer-living species showed greater individual variability in responses (i.e. greater variance) than shorter-living species. Altogether, our results provide some support for the dynamic threshold model of terminal investment, in that reproductive responses to a survival threat vary in an adaptive manner depending on the residual reproductive value.

### Individuals do not terminally invest on average

The lack of an overall effect stands in contrast to a recent qualitative review, which concluded that majority of studies supported the terminal investment hypothesis (i.e. vote-counting) [17]. Our estimate of the overall effect, which was quantitatively derived from a comprehensive dataset of 474 effect sizes from 11,951 individuals (91 papers), was very small. The overall *lnRR* effect size of − 0.03 indicated a mere 3% statistically non-significant difference between the treatment and control group. The same conclusion was corroborated by Hedges’ *d* analysis using an even larger number of effect sizes (see supplementary results; Additional file 3). Therefore, our findings demonstrated very clearly that a terminal investment response is not as common as is thought.

### Large heterogeneity in mean responses across effect sizes

Although the overall effect did not support the main hypothesis, data heterogeneity was very high (*I*^2^ = 96.76%), as expected, with some studies finding an increase in reproductive investment in response to an immune challenge, and other studies finding a decrease. This heterogeneity is unlikely to be driven primarily by a bias towards high profile, significant results in either direction in the literature [55], since we did not find any indication of publication bias from any of our analyses.

The other more theoretically substantive explanation is that individuals terminally invest only under certain circumstances [10]. Recent theoretical models such as the dynamic threshold model recognize such individual variation and have been focused on modelling the circumstances that trigger a terminal investment response [17]. One key prediction is that individuals are more likely to terminally invest the lower their residual reproductive value prior to the immune challenge [17]. Here, we tested the moderating effect of two important indicators of residual reproductive value, age of the sample tested and lifespan of the species. The age class effect provided some support for the dynamic threshold model [17], where older individuals, who are expected to have a lower residual reproductive value, showed a stronger and positive response compared to younger individuals [13, 17].

There are two reasons to interpret the age class effect cautiously. First, we did not find the same effect in Hedges’ *d* analyses. Second, even though we did find a difference between older and younger individuals in the *lnRR* analyses, the terminal investment response in older individuals was not statistically significant when compared against zero. Therefore, the older individuals themselves might not actually be investing terminally. It is possible that even with a non-live immune challenge, individuals still suffer the cost of mounting an immune response, thus limiting the resources available for adjusting reproductive investments (which also explains the heterogeneity in individual responses). It is also possible that the result was non-significant because of the relatively small sample of effect sizes belonging to older individuals (*N*_effect size_ = 33 out of 506), which appear to be coming from mostly insect species, according to our sensitivity analysis on taxonomy. Yet, the *lnRR* of 0.167 indicates that reproductive investment of older individuals increase by an average of 18.1% following an immune challenge. Such level of increase might still be biologically significant despite the lack of statistical significance. We invite future studies on the terminal investment hypothesis to incorporate samples of different ages and a greater range of species/taxonomic groups to test the age class effect further.

Despite our hypothesis, species with shorter lifespans did not show a stronger terminal investment response than longer-living species. One potential criticism of the lifespan results is that we should be examining reproductive lifespan rather than total lifespan. Some species might have an extended non-reproductive developmental stage followed by a short reproductive period (such as insects with extended larval phases) or vice versa, introducing confounds if we use total lifespan as a proxy for reproductive lifespan. However, given the wide range of taxa included in our dataset, with lifespans ranging from 14 to more than 13,000 days, our lifespan data is likely to provide a valid proxy of variation in reproductive lifespan across the species tested. To further improve the validity of our lifespan measure, we statistically controlled for potential confounds due to research efforts for the species in our dataset. We recognize that the correction might not be perfect because the relationship between research effort and lifespan can depend on a number of variables, including the target species or populations (e.g. wild vs lab) [53]. Therefore, caution still needs to be exercised when interpreting the results.

### Individuals respond differently, leading to an increase in variance

The dynamic threshold model predicts not only substantial heterogeneity in mean response across studies, but also that individual responses would cause an increase in variance in the treatment group [17]. Here, by leveraging on recent developments in the meta-analysis of changes in variance between groups [23], our results provided novel support for this important theoretical prediction that has been neglected to date. The overall *lnCVR* effect size indicated that following an immune challenge, treatment groups responded with an average 12% increase in variance over the control groups. This finding is consistent with other recent meta-analyses, where individuals exposed to stressful environments demonstrated increased variance in their responses relative to the control group. Examples include dietary and temperature stress [19, 20, 51]. Furthermore, changes in variance are unlikely to be subject to publication biases as they have not been the explicit focus of most scientific hypotheses until recently [51]. Therefore, we are inclined to believe that this increase in variance represents a genuine biological effect. However, we do urge some caution, as the *lnSD* result did not reach significance, even though it was in the same direction as the *lnCVR* result.

### Degree of change in variance varies with lifespan of species

Besides the average increase in variance, there was also substantial heterogeneity in the effect sizes (*I*^2^ = 67.94%), indicating that the variance changes differed across studies. Part of this heterogeneity was explained by the lifespan of the species tested. As hypothesized, longer-living species showed a greater increase in the variance of their reproductive investment responses to an immune challenge. Subset analysis comparing the two largest taxonomic groups in our dataset, namely birds and insects, showed that this effect is likely due to variations both between and within taxonomic groups (for birds). Not only did we find that birds, which are longer-living on average, had a stronger variance response than insects, but we also found that within bird species, lifespan positively predicted the degree of variance change. In general, longer-living species have greater phenotypic plasticity [1]. In the case of reproduction, a longer lifespan often translates to a greater number of reproductive opportunities and reproductive events [1]. Therefore, relative to a shorter-living species, individuals from a longer-living species are more likely to show varying responses to an immune challenge, depending on how close they are to their terminal investment threshold [17]. Differences in lifespan can also lead to important differences in the immune system between taxa [56]. A greater variance response in birds might arise from them having a more sophisticated immune system that comprises both innate and adaptive immunity, as compared to insects, which only possess innate immunity. The results here are consistent with the theoretical idea that the individual reproductive investment responses represent an adaptive plastic response that has evolved for maximizing fitness [57, 58]. They also demonstrate that changes in variance provide a useful way to test for such individual plastic responses. Therefore, we urge researchers to pay more attention to changes in variance in future studies.

### Limitations and future directions

We were only able to identify a small number of moderators that influenced the meta-analytic results, two for the mean response and one for the variance response. Therefore, much of the heterogeneity in effect sizes remains unaccounted for. One difficulty we faced was that multiple potential moderators, including taxonomic group, parental care, and experimental setting, had to be dropped from the analyses, due to multicollinearity. Clearly, much remains to be understood concerning the factors that drive variation in reproductive investment responses when individuals are faced with a mortal threat. We encourage researchers to delve further into studying factors that impact the residual reproductive value, such as age, the dosage of immune challenges, and individual quality (e.g. challenging individuals that have been artificially selected for individual quality).

## Conclusions

Across the studies identified in our systematic search, individuals did not consistently upregulate their reproductive investment when faced with a survival threat. We found substantial individual variation in responses, by looking at both the heterogeneity in effect sizes for the mean results and by meta-analysing the changes in variance between the immune challenge and control groups. Furthermore, consistent with theoretical predictions, we found that these variations are accounted for by factors that are linked to the residual reproductive value of individuals, such as age class of the sample (for heterogeneity in mean effects) and lifespan of the species (for degree of changes in variance post-challenge). Overall, our result provides some support for the dynamic threshold model, in that a terminal investment response is nuanced and likely to occur only when the residual reproductive value of the individual is low.

## Supplementary Information

**Additional file 1.** Raw Data.

**Additional file 2.** R analysis code.

**Additional file 3: Supplementary results.** [Hedges’ d and lnSD results]. **Table S1.** [Heterogeneity by random effects]. **Table S2.** [Hedges’ d results]. **Table S3.** [lnRR models’ AICc]. **Table S4.** [lnCVR models’ AICc]. **Table S5.** [Hedges’ d AICc]. **Figure S1.** [Association among moderators]. **Figure S2.** [Orchard plots for lnRR without truncation]. **Figure S3.** [Relationship plots for lnRR without truncation]. **Figure S4.** [Orchard plots for lnCVR without truncation]. **Figure S5.** [Relationship plots for lnCVR without truncation].

## Data Availability

The data and R analysis code are provided as electronic supplementary material.

## Abbreviations

PRISMA-P: Preferred Reporting Items for Systematic Review and Meta-Analysis Protocols
PICO: Population, Intervention, Comparison/Control group, Outcome
*lnRR*: Natural logarithm of the response ratio
*lnCVR*: Natural logarithm of the ratio between the coefficients of variation
*lnSD*: Natural logarithm of the standard deviation
lnMean: Natural logarithm of mean
CI.lb: Lower bound of the confidence interval
CI.ub: Upper bound of the confidence interval
