# Supplementary material for: The influence of immune challenges on the mean and variance in reproductive investment: a meta-analysis of the terminal investment hypothesis
Source: BMC Biol. 2023 May 12;21:107. doi: 10.1186/s12915-023-01603-4 (PMC10176797; doi:10.1186/s12915-023-01603-4)
Supplement: Supplementary file 3 — Additional file 3: Supplementary results. [Hedges’ d and lnSD results]. Table S1. [Heterogeneity by random effects]. Table S2. [Hedges’ d results]. Table S3. [lnRR models’ AICc]. Table S4. [lnCVR models’ AICc]. Table S5. [Hedges’ d AICc]. Figure S1. [Association among moderators]. Figure S2. [Orchard plots for lnRR without truncation]. Figure S3. [Relationship plots for lnRR without truncation]. Figure S4. [Orchard plots for lnCVR without truncation]. Figure S5. [Relationship plots for lnCVR without truncation]. [file 12915_2023_1603_MOESM3_ESM.docx]

**Supplementary material**

The influence of immune challenges on the mean and variance in reproductive investment: a meta-analysis of the terminal investment hypothesis

Yong Zhi Foo^1^, Malgorzata Lagisz^1^, Rose E. O’Dea^1^, Shinichi Nakagawa^1^

^1^Evolution & Ecology Research Centre, School of Biological and Environmental Sciences, University of New South Wales, Sydney, 2052 NSW, Australia

Emails: fooyongzhi@gmail.com, losialagisz@gmail.com, rose.eleanor.o.dea@gmail.com, itchyshin@gmail.com

**Supplementary results**

**Effect of immune challenge on mean reproductive investment response using Hedges’ *d***

Overall, there was no effect of an immune challenge on mean reproductive investment, *lnRR* = -0.09, *p* = 0.10, 95% CI (-0.19, 0.02) (Supplementary Table 2). Among the five potential random effects shown in Supplementary Table 1, paper ID and observation ID accounted for a substantial proportion of the heterogeneity and were therefore retained in all meta-analytic models testing the mean response.

**Supplementary Table 1.** Heterogeneity among the mean effect sizes by potential random effects.

|  | *I^2^* |
| --- | --- |
| Total | 82.06% |
| Paper ID | 47.69% |
| Study ID | 2.52% |
| Observation ID | 30.40% |
| Species ID | 2.09% |
| Phylogeny | 0.00% |

Given the large heterogeneity among the effect sizes, *I^2^* = 77.71%, we conducted moderator analyses. The final averaged model from the AICc model selection included five moderators: reproductive category, year of publication, blinding, age class, and control procedure (Supplementary Table 2). Out of these moderators, only reproductive category showed an effect, with reproductive output showing a negative effect (Supplementary Table 2) that was significantly different from offspring traits, estimate = 0.27, *p* = 0.007, 95% CI (0.07, 0.47) and behavioural traits, estimate = 0.19, *p* = 0.03, 95% CI (0.01, 0.37). Unlike the *lnRR* results, we did not find a significant age class effect, despite observing the same trend that older individuals had larger positive effect than younger individuals, estimate = -0.18, *p* = 0.08, 95% CI (-0.37, 0.02). The other three moderators did not reveal any statistically significant effects (*p*-values ranging from 0.29 to 0.42).

**Supplementary Table 2.** Parameter estimates, *p*-values, and marginal *R*^2^ for the effect of an immune challenge on mean reproductive investment. M is the mean Hedges’ *d* effect size (positive value indicates increased reproductive investment for the treatment group), CI.lb and CI.ub are the lower and upper bounds of the 95% confidence interval.

|  | M | P | CI.lb | CI.ub | marginal *R*^2^ |
| --- | --- | --- | --- | --- | --- |
| **Meta-analytic mean** | -0.088 | 0.098 | -0.193 | 0.017 |  |
| **Age class** |  |  |  |  |  |
| Unclear/Mixed | -0.045 | 0.577 | -0.202 | 0.113 | 0.009 |
| Old | 0.062 | 0.478 | -0.110 | 0.233 |  |
| Young | -0.126 | 0.111 | -0.281 | 0.029 |  |
| **Control procedure** |  |  |  |  |  |
| No | -0.126 | 0.163 | -0.305 | 0.052 | 0.002 |
| Yes | -0.065 | 0.183 | -0.162 | 0.031 |  |
| **Source of animals** |  |  |  |  |  |
| Cultured population | -0.107 | 0.306 | -0.314 | 0.099 | 0.001 |
| Wild or immediate offspring of wild | -0.062 | 0.412 | -0.210 | 0.086 |  |
| Wild-caught but kept in research facilities for generations | -0.082 | 0.462 | -0.300 | 0.137 |  |
| **Reproductive investment categories** |  |  |  |  |  |
| Behavioural traits | 0.017 | 0.893 | 0.838 | -0.146 | 0.033 |
| Offspring traits and success | 0.088 | 0.517 | 0.503 | -0.172 |  |
| Others | -0.144 | 0.196 | 0.216 | -0.373 |  |
| Physiological/Physical traits | -0.099 | 0.180 | 0.287 | -0.283 |  |
| Reproductive output | -0.190 | 0.008 | 0.010 | -0.333 |  |
| **Immune challenge type** |  |  |  |  |  |
| Non-pathogenic foreign bodies | -0.050 | 0.803 | -0.441 | 0.342 | 0.000 |
| Substrates of pathogenic origins | -0.088 | 0.108 | -0.195 | 0.020 |  |
| **Blinding** |  |  |  |  |  |
| No/Unclear | -0.112 | 0.056 | -0.226 | 0.003 | 0.015 |
| Yes | 0.063 | 0.574 | -0.158 | 0.283 |  |
| **Incomplete reporting** |  |  |  |  |  |
| No | -0.075 | 0.213 | -0.194 | 0.044 | 0.000 |
| Yes | -0.097 | 0.408 | -0.328 | 0.134 |  |
| **Log(lifespan of species) (mean centered and controlling for research effort for that species)** | 0.019 | 0.750 | -0.101 | 0.139 | 0.002 |
| **Journal impact factor (mean centered)** | 0.012 | 0.768 | -0.067 | 0.091 | 0.000 |
| **Year of publication (mean centered)** | -0.068 | 0.226 | -0.179 | 0.043 | 0.014 |

**Effect of immune challenge on variance in reproductive investment response using *lnSD***

Our *lnSD* results did not agree with the *lnCVR* results. Overall, there was no effect of an immune challenge on variance in reproductive investment, estimate = 0.01, *p* = 0.78, 95% CI (-0.07, 0.09).

**Supplementary tables**

**Supplementary Table 3.** AICc values of models that were included in the final averaged moderator model for *lnRR.*

| intercept | blinding | incomplete reporting | age class | reproductive categories | treatment type | publication year | df | logLik | AICc | delta | weight |
| --- | --- | --- | --- | --- | --- | --- | --- | --- | --- | --- | --- |
| + |  |  | + |  |  |  | 6 | -269.85 | 551.90 | 0.00 | 0.13 |
| + |  |  | + |  |  | -0.038 | 7 | -268.86 | 551.90 | 0.07 | 0.12 |
| + |  |  | + | + |  |  | 10 | -265.98 | 552.40 | 0.54 | 0.10 |
| + |  |  | + |  | + |  | 7 | -269.34 | 552.90 | 1.02 | 0.08 |
| + |  |  | + | + |  | -0.034 | 11 | -265.20 | 552.90 | 1.06 | 0.07 |
| + |  |  | + |  | + | -0.037 | 8 | -268.42 | 553.10 | 1.25 | 0.07 |
| + |  |  |  | + |  |  | 8 | -268.48 | 553.30 | 1.39 | 0.06 |
| + | + |  | + |  |  | -0.040 | 8 | -268.54 | 553.40 | 1.51 | 0.06 |
| + |  |  |  |  |  |  | 4 | -272.68 | 553.40 | 1.58 | 0.06 |
| + |  |  |  |  |  | -0.038 | 5 | -271.67 | 553.50 | 1.59 | 0.06 |
| + | + |  | + |  |  |  | 7 | -269.64 | 553.50 | 1.64 | 0.06 |
| + |  |  | + | + | + |  | 11 | -265.56 | 553.60 | 1.77 | 0.05 |
| + |  | + | + |  |  |  | 7 | -269.78 | 553.80 | 1.91 | 0.05 |
| + |  |  |  | + |  | -0.033 | 9 | -267.74 | 553.80 | 1.97 | 0.05 |

**Supplementary Table 4.** AICc values of models that were included in the final averaged moderator model for *lnCVR*.

| intercept | control procedure | incomplete reporting | treatment type | lifespan | research effort | df | logLik | AICc | delta | weight |
| --- | --- | --- | --- | --- | --- | --- | --- | --- | --- | --- |
| + |  |  |  | 0.086 | 0.012 | 7 | -313.921 | 642.1 | 0.000 | 0.243 |
| + | + |  |  | 0.093 | 0.015 | 8 | -313.110 | 642.6 | 0.450 | 0.194 |
| + |  |  |  |  |  | 5 | -316.476 | 643.1 | 0.990 | 0.148 |
| + |  | + |  | 0.090 | 0.013 | 8 | -313.635 | 643.6 | 1.500 | 0.115 |
| + | + |  |  |  |  | 6 | -315.769 | 643.7 | 1.630 | 0.107 |
| + |  |  | + | 0.085 | 0.011 | 8 | -313.774 | 643.9 | 1.780 | 0.100 |
| + | + | + |  | 0.097 | 0.015 | 9 | -312.800 | 644.0 | 1.920 | 0.093 |

**Supplementary Table 5.** AICc values of models that were included in the final averaged moderator model for Hedges’ *d*.

| intercept | blinding | control procedure | reproductive category | age class | publication year | df | logLik | AICc | delta | weight |
| --- | --- | --- | --- | --- | --- | --- | --- | --- | --- | --- |
| + |  |  | + |  |  | 7 | -566.96 | 1148.10 | 0.00 | 0.24 |
| + |  |  | + | + |  | 9 | -565.37 | 1149.10 | 0.93 | 0.15 |
| + | + |  | + |  |  | 8 | -566.47 | 1149.20 | 1.06 | 0.14 |
| + |  |  | + |  | -0.05 | 8 | -566.47 | 1149.20 | 1.08 | 0.14 |
| + |  | + | + |  |  | 8 | -566.64 | 1149.50 | 1.42 | 0.12 |
| + | + |  | + |  | -0.06 | 9 | -565.80 | 1149.90 | 1.80 | 0.10 |
| + | + |  | + | + |  | 10 | -564.80 | 1150.00 | 1.87 | 0.10 |

**Supplementary figures**

**Supplementary figure 1.** Goodman and Kruskal’s τ measure of association between categorical moderator variables. The tests show strong associations among four of the moderators: taxonomic group, parental care, lifespan, and experimental setting, where birds and mammals were more likely to be long living and to provide parental care. Birds were also more likely to be studied in the wild. Note: lifespan was recoded into a categorical variable of short (<2,000 days) and long (>2,000 days) lifespan species in order to run the association tests with the other categorical moderators.


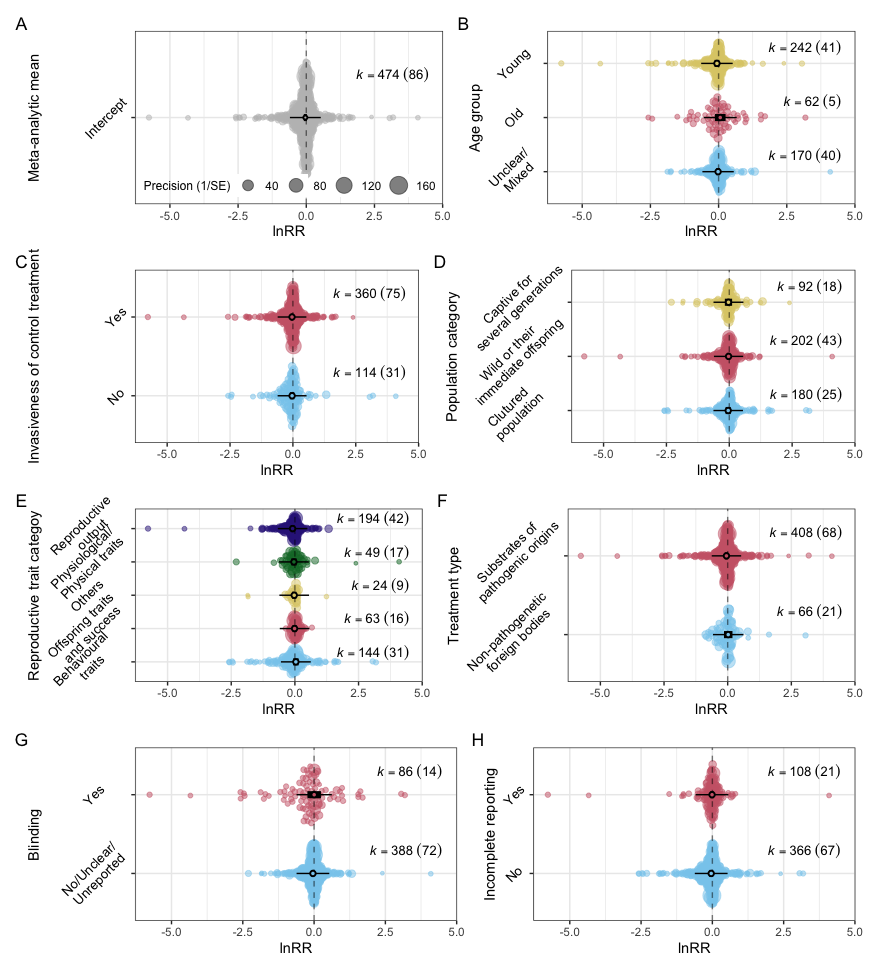


**Supplementary figure 2.** Orchard plots [53] for the (A) overall effect size and (B-H) categorical moderator effects for mean reproductive investment responses to an immune challenge. Positive *lnRR* indicates increased reproductive investment in the treatment group. Each plot includes the mean effect size (open circle), 95% confidence interval (thick error bars around the mean effect size), 95% prediction interval (thin error bars), and the distribution of individual effect sizes (with the size of the points corresponding to their precision). *k* refers to the number of effect sizes and number of studies in parenthesis.


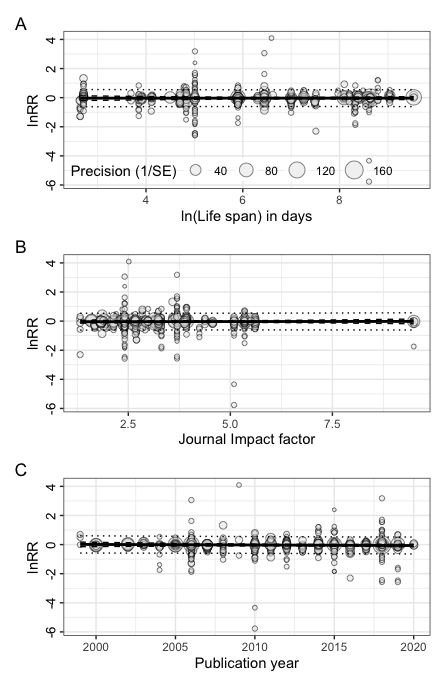


**Supplementary figure 3.** Relationship between effect size and lifespan, publication journal impact factor, and publication year for mean reproductive investment responses to an immune challenge. Size of each point corresponds to the precision (inverse of standard error).


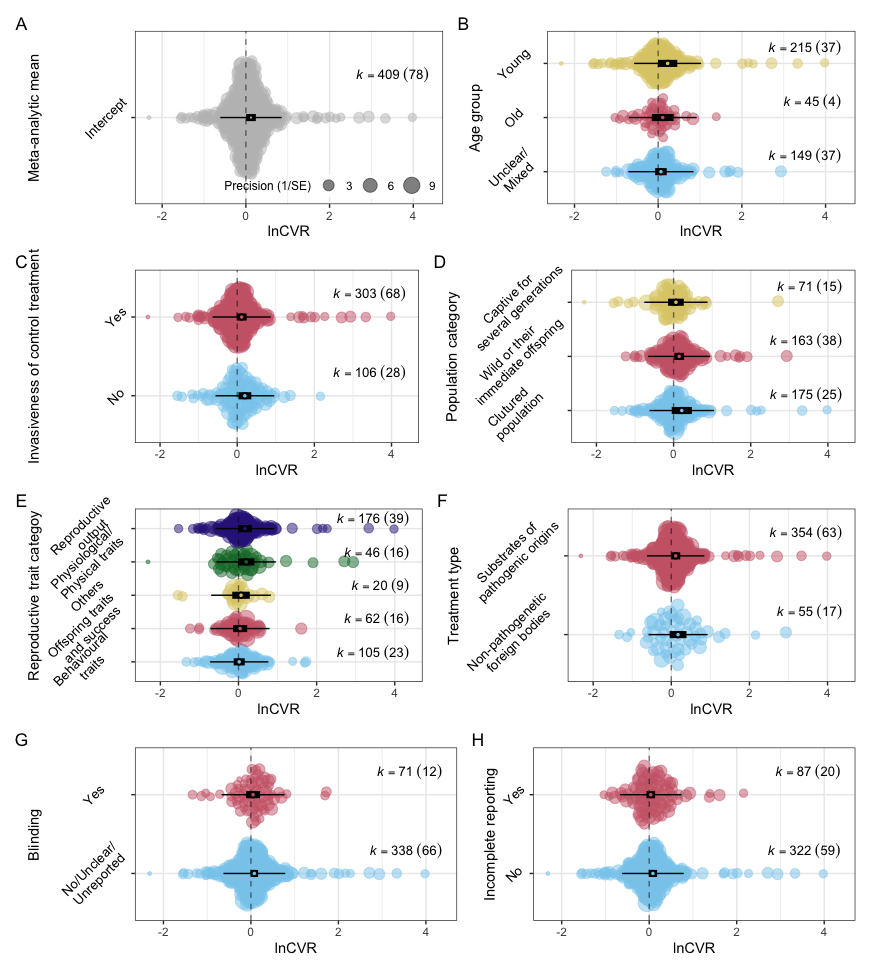


**Supplementary figure 4.** Orchard plots for the (A) overall effect size and (B-H) categorical moderator effects for variance in reproductive investment responses to an immune challenge. Positive *lnCVR* indicates increased variance in the treatment group. Each plot includes the mean effect size (open circle), 95% confidence interval (thick error bars around the mean effect size), 95% prediction interval (thin error bars), and the distribution of individual effect sizes (with the size of the points corresponding their precision). *k* refers to the number of effect sizes and number of studies in parenthesis.


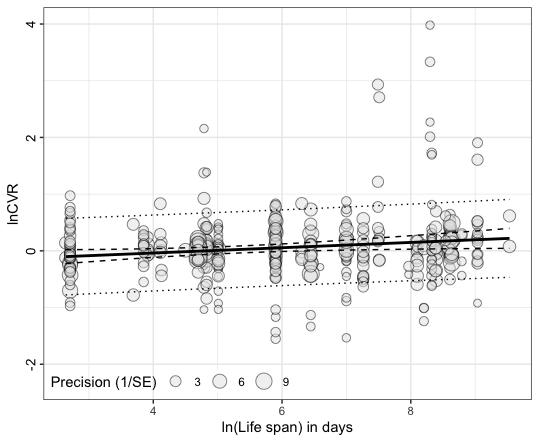


**Supplementary figure 5.** Relationship between effect size and lifespan for variance in reproductive investment responses to an immune challenge. Size of each point corresponds to the precision (inverse of standard error).
